# Influence of the pelvic bone and gluteal zone over the stress state at the interaction between the prosthetic socket and residual limb for people with transfemoral amputation

**DOI:** 10.3389/fbioe.2026.1938922

**Published:** 2026-09-30

**Authors:** Juan Atehortua, Fanny Valencia, Juan Ramírez

**Affiliations:** 1 Department of Mechanical Engineering, Universidad Nacional de Colombia, Medellin, Colombia; 2 Department of Physical Therapy, María Cano University Foundation, Medellín, Colombia

**Keywords:** explicit finite element analysis (FEA), finite element model, pelvic bone, people with transfemoral amputation, soft tissue

## Abstract

The rehabilitation process for people with lower limb amputation represents a substantial socioeconomic, psychological, and functional challenge. Finite element analysis (FEA) has been increasingly used to investigate residual limb–socket interaction; however, the extent to which additional proximal anatomy, beyond the anatomical structures commonly included in simplified models, is necessary to adequately represent the mechanical response remains unclear. This study aimed to determine whether incorporating additional proximal anatomy, specifically the gluteal soft tissue and pelvic bone, produces mechanically significant changes in FEA results compared with simplified models that include only the femur, partial residual soft tissue, and prosthetic socket, while considering the associated increase in model complexity and computational cost. Information from 15 patients with unilateral transfemoral amputation was used to develop 45 patient-specific numerical models, comprising three anatomical configurations: Model 1 included the socket, femur, and residual soft tissue without the gluteal region; Model 2 additionally incorporated the gluteal soft tissue; and Model 3 further incorporated the pelvic bone. The models preserved each patient’s actual socket geometry, with quadrilateral, semi-containment, and exomodular socket designs represented across the study population. The analysis focused on the residual limb–socket interaction. The results showed that incorporating the gluteal region and pelvic bone did not produce statistically significant differences in the magnitude of the mean contact stress (CPRESS during the gait cycle stage for Model 1: 29.757 kPa for Model 2: 32.183 kPa and for Model 3: 31.853 kPa), although changes in the spatial distribution of the stress state were observed. These findings indicate that the inclusion of additional proximal anatomy may have limited influence on global contact-stress magnitude while affecting local stress distributions. Therefore, the relevance of incorporating the pelvis and gluteal region should be considered in relation to the specific objective of the simulation and the additional computational cost. This study does not evaluate the biomechanical effect of ischial support or compare different socket designs for the same patient; rather, it evaluates the mechanical implications of increasing anatomical model fidelity while maintaining each patient’s specific socket configuration.

## Introduction

Globally, the main causes of lower limb amputation (LLA^1^) include vascular diseases, infections, congenital malformations, trauma, and diabetes, the last the major cause. The rehabilitation process for people with LLA represents a substantial socioeconomic, psychological, and functional challenge (Ramírez, 2011; Dickinson et al., 2017). The main objective of prosthetic systems is to restore and improve the quality of life (QOL^2^) of their users (Dickinson et al., 2017; (Safari, 2020; Tyagi et al., 2024). The interface between the residual limb and the socket is the place where skin damage most commonly occurs during the rehabilitation process, which is mainly caused by inadequate socket design (Dickinson et al., 2017) The finite element method (FEM^3^) has been extensively used as a tool to evaluate the stress state of the residual limb while interacting with the socket for LLA, which would aid in the more efficient and effective design of prosthetic systems (Dickinson et al., 2017; Portnoy et al., 2008; Lee et al., 2005; Lacroix and Ramírez Patiño, 2011; Henao et al., 2020a; Lee et al., 2004; Roberts et al., 2020).

The development of numerical models for residual limbs of people with transfemoral amputation (PTA^4^) has focused primarily on people with transtibial amputation (Portnoy et al., 2008; Lee et al., 2005; Lee et al., 2004; Zhang et al., 1995; Wu et al., 2003; Lin et al., 2004; Jia et al., 2004; Portnoy et al., 2009; Ballit et al., 2020; Steer et al., 2020; Cagle et al., 2018). Since 2010, the interest in developing numerical models for PTA has been growing (Dickinson et al., 2017). However, in 1996, M. Zhang et al. (Zhang and Mak, 1996) conducted the first two-dimensional FEM study on the residual limb of PTA to understand its mechanical behavior while interacting with the socket. Thereafter, Lacroix et al. (Lacroix and Ramírez Patiño, 2011) conducted their study on the first approximation of the FEM of PTA in three dimensions, where a suitable model representing the socket donning process and a dynamic representation of the gait cycle was also defined, making the numerical model more realistic.

Furthermore, three-dimensional (3D) numerical models have been developed for PTA. Zhang et al. (Zhang et al., 2013) took into account the coefficients of friction (COF^5^) in this type of models; however, the simulation protocol defined did not consider the donning and stabilization steps, and the load was considered static. Thereafter, the study by Ramírez et al. (Ramírez et al., 2015) defined the COF between the residual limb and the socket for PTA in the two situations occurring during the gait cycle: one considering the absence of sweat and the other considering the presence of sweat and residual limb hair.

Subsequent studies on the simulation process for PTA models had included a contact condition between the soft tissue and the socket during the donning process according to literature reports, as well as present variations in the loading condition; however, all such studies only included a portion of the soft tissue that does not consider the gluteal zone or the pelvic bone (Henao et al., 2020a; Steer et al., 2020; Restrepo et al., 2014; Vélez et al., 2016; Mejía-Blandón et al., 2018; Ramírez and Vélez, 2012; Meng et al., 2020; Zhong et al., 2023). However, while the study by Heesewijk et al. (van Heesewijk et al., 2018) did include the pelvic bone, the simulation process defined and the process of obtaining the model geometries were not adequate, leading to inaccurate results.

Many studies based on the FEM for LLA have used linear elastic models to represent the mechanical behavior of soft tissue (Lee et al., 2005; Lee et al., 2004; Zhang et al., 1995; Wu et al., 2003; Lin et al., 2004; Jia et al., 2004; Zachariah and Sanders, 2000). Nevertheless, different models have been proposed to represent the mechanical behavior of soft tissue. For instance, the studies by Portnoy et al. (Portnoy et al., 2008; Portnoy et al., 2009) applied the viscoelastic models. Moreover, studies over the last decade have investigated the mechanical behavior of soft tissues using numerical models of the lower limb with hyperelastic models (Dickinson et al., 2017; Safari, 2020; Henao et al., 2020a; Restrepo et al., 2014; Vélez et al., 2016; Ramírez and Vélez, 2012; Isaza et al., 2019; Isaza et al., 2013). In all such studies, soft tissue had been defined as a bulk representing skin, fat, and muscle; therefore, the bulk was characterized as having a unique mechanical behavior.

Considering the previously facts and the need to develop more accurate and realistic numerical models, the current study aimed to determine whether the gluteal zone of the residual limb influenced the state and distribution stress in the interaction between the residual limb and the socket among people with transfemoral amputation.

## Methods

This study used information from 15 patients with unilateral transfemoral amputation. Before the acquisition and use patient information, all patients provided written informed consent in accordance with the guidelines set by the Ethics Committee of Universidad Nacional de Colombia. The process by which the necessary information for this study was obtained is detailed in a previous study *(Nivel de Confort y Distribución de Esfuerzos en la Interfaz Socket-Muñón en Amputados Transfemorales* (Ramírez, 2011).

The patients included in the study used different types of prosthetic sockets, including quadrilateral, semi-containment, and exomodular designs. Each patient was modeled using the specific socket geometry corresponding to their individual prosthetic configuration.

Table 1 shows the general information of each participant included in this study. All subjects used the prosthetic system in their daily lives and did not have associated comorbidities such as vascular, neurological, or psychological conditions that may alter or modify the results of the numerical model simulation.

**TABLE 1:** General information of the patients with transfemoral amputation.

Patient N°*	Time since amputation (year)*	Weight without prosthesis (kg)	Prosthesis weight (kg)	Socket type	Height (cm)	Sound leg length (cm)	Residual limb length (cm)
P1-L	9	72	5	Quadrilateral	176	86.5	28
P2-R	2	57	4.1	Quadrilateral	172	80.5	21
P3-L	7	78	3.6	Quadrilateral	169	74	22
P4-L	7	91.5	4.9	Quadrilateral	177	86	23
P5-R	2	75.3	2.9	Quadrilateral	168	76.5	36.5
P6-R	4	67.9	3.2	Semi-containment	175	85	30
P7-L	8	70	3	Semi-containment	171	85	33
P8-L	24	73.6	6.2	Exomodular	167	79	20
P9-L	6	72	4.1	Quadrilateral	167	80	19
P10-L	28	67.7	5	Semi-containment	170	83	21
P11-R	42	64.5	3.7	Semi-containment	161	77	10
P12-L	23	64.7	2.8	Quadrilateral	164	83	28
P13-L	4	81	6.6	Quadrilateral	171	85	31
P14-R	3	59	4.6	Quadrilateral	162	78	17
P15-R	5	59	4	Semi-containment	168	83	24

* In the Patient No. Column, the letter R or L followed by the patient number indicates whether the amputated limb is the right or left limb, respectively.

### Simulation process

Three numerical models were developed for each patient. The first model (M1) includes the socket, femur, and residual soft tissue without the gluteus zone. The second model (M2) includes the socket, femur, and residual soft tissue with the gluteus zone. The third model (M3) includes the socket, femur, pelvic bone, and residual soft tissue with the gluteus zone (see Figure 1).

**FIGURE 1:**
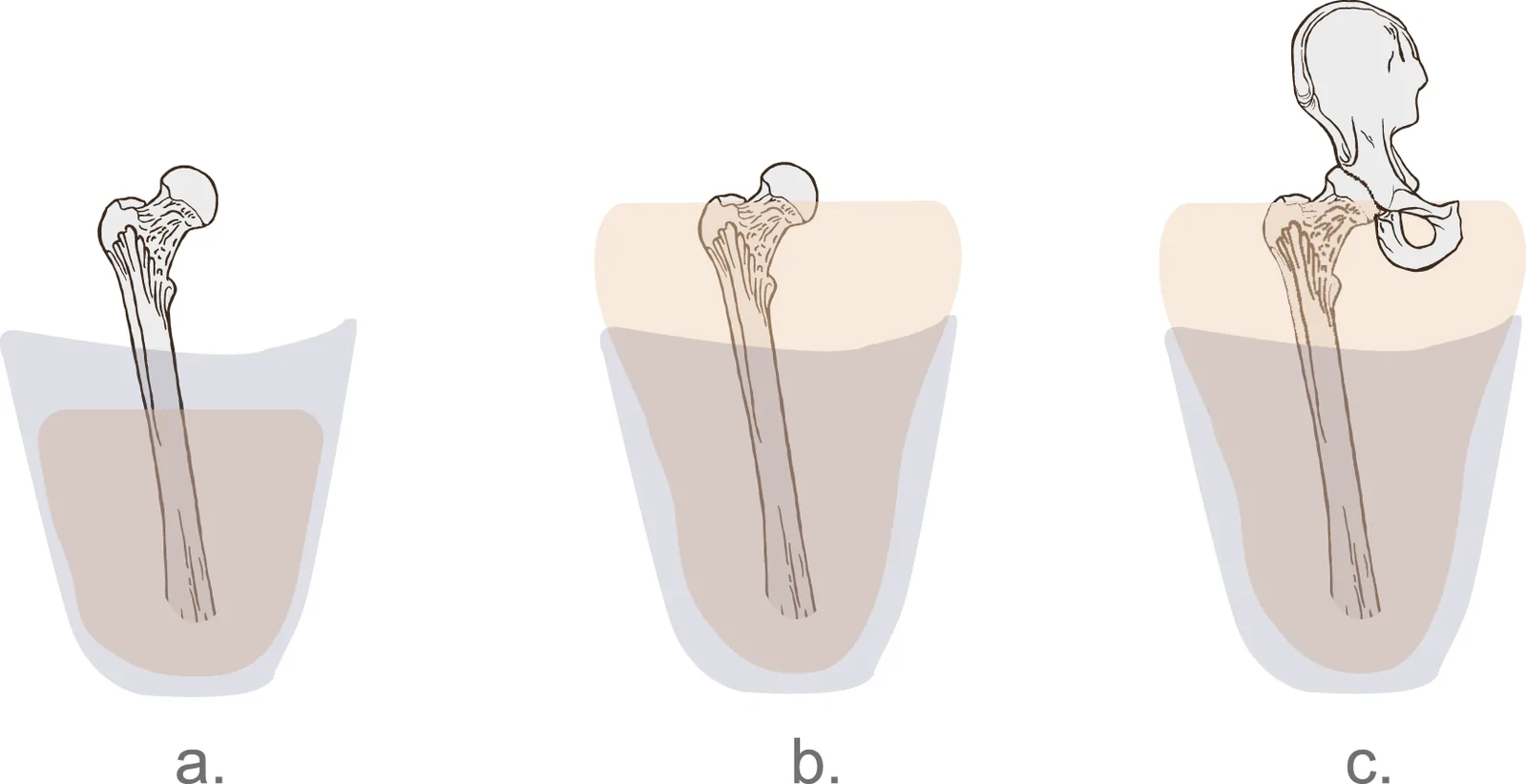
Types of numerical models developed. Model 1 includes the femur, socket, and residual soft tissue without the gluteal zone **(a)**. Model 2 includes the femur, socket, and residual soft tissue with a portion of the gluteal zone **(b)**. Model 3 includes the femur, pelvic bone, socket, and residual soft tissue with a portion of the gluteal zone **(c)**.

Figure 2 shows the simulation process for each model. All models were developed in three sequential steps. The first step represents the socket donning (a), the second step represents the stabilization period (b), and the final step represents the gait cycle in the maximum load condition. All models were dynamic, and the information used to represent the gait cycle (A single maximum-loading time point with a duration of 0.1 s) in the current work was obtained from a previous study (Lacroix and Ramírez Patiño, 2011) using the inverse dynamics technique and data obtained during the motion analysis of each patient.

**FIGURE 2:**
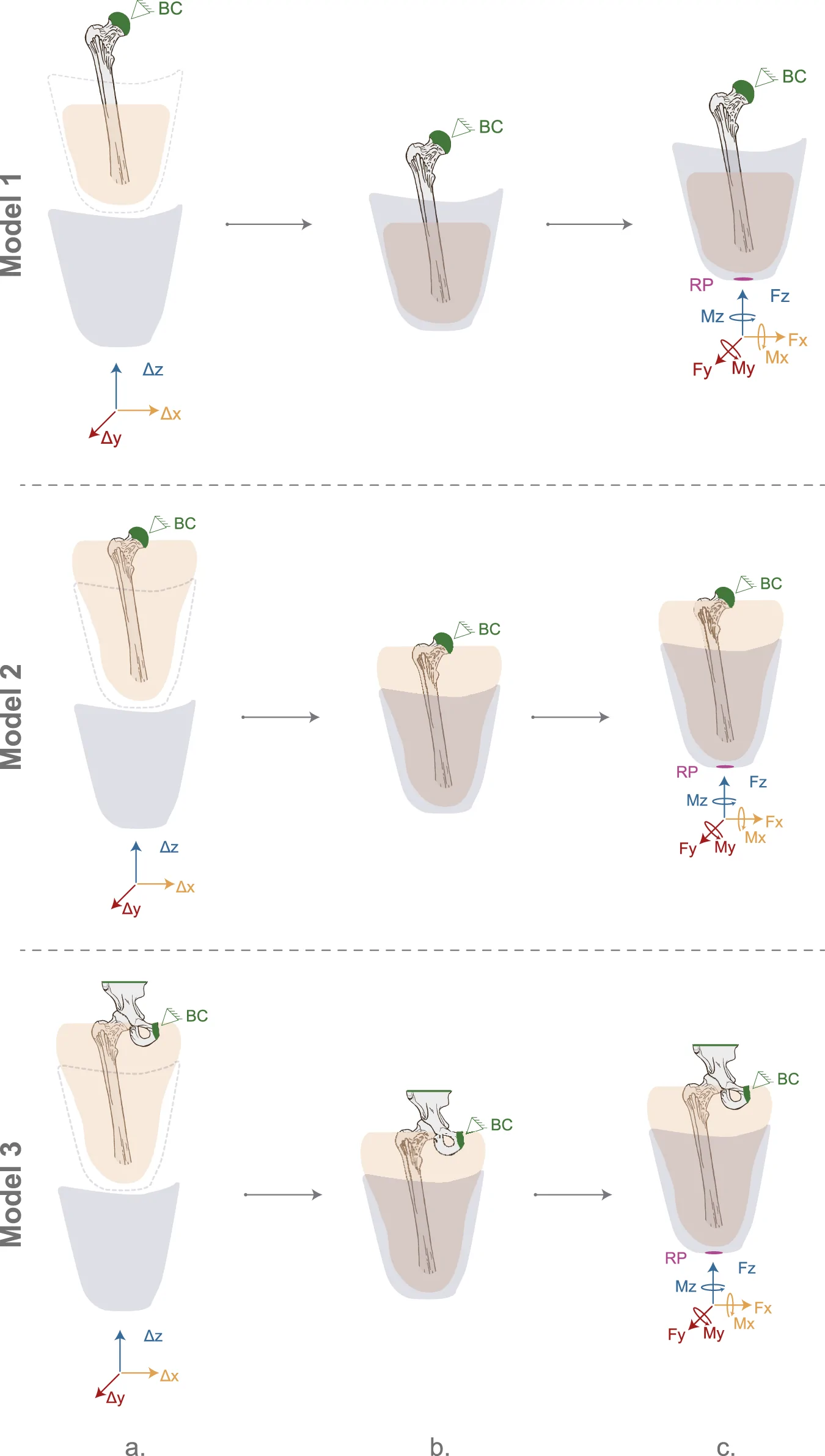
Simulation process and free-body diagram for models 1, 2, and 3. Types of numerical models developed. Model 1 includes the femur, socket, and 121 residual soft tissue without the gluteal zone **(a)**. Model 2 includes the femur, socket, 122 and residual soft tissue with a portion of the gluteal zone **(b)**. Model 3 includes the 123 femur, pelvic bone, socket, and residual soft tissue with a portion of the gluteal zone **(c)**.

### Geometry

Owing to the complex shape, all geometries used to develop the numerical models in this study were obtained individually using different techniques to ensure accuracy. The socket and residual soft tissue without the gluteus zone were reconstructed from a cast model for each patient with the help of a 3D scanner (NextEngine Inc., Santa Monica. United States). The femur and pelvic bone for the residual limb were obtained from the 3D reconstruction using the computerized axial tomography information with Mimics 16.0 (Materialise, Leuven, Belgium), a software that allows for medical image segmentation. The process to assembly all elements was conducted using the gait cycle simulation free software *OpenSim* and the inverse dynamic algorithm by Rajagopal et al. (Rajagopal et al., 2016) and validated using the reports by Jacquelin et al. (Perry, 1992) to ensure that the orientation of the pelvic bone with respect to the femur correctly represents the gait cycle phase in its maximum load condition. Given that information of soft tissue reconstruction available did not include the gluteus zone, the gluteus zone geometry for models 2 and 3 was determined based on a projection of the residual limb reconstructed without the gluteus zone for the model 1. This approach was adopted because the gluteal region may be deformed by contact with the imaging surface during CT acquisition, and therefore may not represent its unloaded anatomical configuration. The projection of the soft-tissue geometry representing the gluteal region was performed from the most proximal portion of the residual limb that was not affected by deformation. Although scars in residual limbs of TA play an important role in the development of numerical models (Portnoy et al., 2009), this geometry was not included in this study given that our main aim was the development of a numerical simulation of the interface between the residual limb and the socket. Detailed information on the tomography parameters, geometry reconstruction, and donning procedures followed in this study can be found in Lacroix and Ramirez (Lacroix and Ramírez Patiño, 2011).

### Materials

To define the mechanical behavior of each component involved in these models, literature reports for studies of this type were considered (Dickinson et al., 2017; Lacroix and Ramírez Patiño, 2011; Henao et al., 2020a; Lee et al., 2004; Wu et al., 2003; Jia et al., 2004; Zhang et al., 2013; Vélez et al., 2016; Ramírez and Vélez, 2012; van Heesewijk et al., 2018). The socket, which was made of polypropylene, had a linear elastic homogeneous and isotropic behavior, with Young’s modulus set at 1.5 GPa and Poisson’s ratio set to 0.3 (Dickinson et al., 2017; Lacroix and Ramírez Patiño, 2011; Lee et al., 2004; Zhang and Mak, 1996). The pelvic bone and femur had a linear elastic homogeneous and isotropic behavior, with Young’s modulus set at 15 GPa and Poisson’s ratio at 0.3 (Ramírez, 2011; Dickinson et al., 2017; Lacroix and Ramírez Patiño, 2011; Henao et al., 2020a; Steer et al., 2020; Duchemin et al., 2008). Due to the highest strains of the soft tissues, a hyperelastic behavior was defined for this simulation. The behavior was defined using the generalized *Neo-Hookean* strain energy potential equation (Lacroix and Ramírez Patiño, 2011; Henao et al., 2020a; Restrepo et al., 2014; Duchemin et al., 2008; Tran et al., 2007; Henao et al., 2020b; Henao et al., 2020c; Geerligs, 2010; Avril et al., 2010; Simulia, 2012; Isaza López and Ramírez Patiño, 2013):

$$W=C_{10}\left(I_{1}-3\right)+\frac{1}{D_{1}}{\left(J-1\right)}^{2}$$

where the invariants of the principal stretch ratios are: $I_{1}=\lambda_{1}^{-2}+\lambda_{2}^{-2}\;\lambda_{3}^{-2}$ . The relative volume change is J , while $C_{10}\;y\;D_{1}$ are the constitutive parameters. For this study, $C_{10}=0.0116\text{ MPa and }D_{1}=11.9\;{\text{MPa}}^{-1}$ were used (Tran et al., 2007; Henao et al., 2020b; Isaza López and Ramírez Patiño, 2013; Affagard et al., 2015).

### Boundary conditions

For models 1 and 2, a boundary conditions (BC^6^) that constrains the displacements and rotations on the femur head was defined. For model 3, a tie constrain was defined between the pelvic bone and femur, and pelvic bone displacement and rotation were restricted, specifically in the iliac crest zone and the connection with the sacrum bone. The boundary conditions (BCs) of Model 3 ensure an equivalent equilibrium system to Models 1 and 2. Therefore, the stress state can be directly compared across the three models, despite the inclusion of an additional component (the pelvis).

Given the complexity of this type of models, a tetrahedral finite element type was used. The element size was between 2.5 and 3 mm. The total number of elements was approximately between 300,000 and 600,000. Depending on the patient, the routine lasted between 9 and 24 h using a Xeon E5-1,650 v2 with a 3.50 GHz processor and 64 GB of RAM on a 64-Bit Windows 10 system. All models were developed in ABAQUS V6.12-3 (Dassault Systèmes SolidWorks Corp., Waltham, United States) in the explicit modulus.

### Contact

The interaction between the residual limb and femur was modeled as a tie constrain, which represented the non-relative movement between these parts. The interaction between the socket and the residual limb was defined using the surface-to-surface contact condition in ABAQUS. This condition prevents the nodes from the slave surface (residual limb mesh) from penetrating into the nodes of the master surface (socket mesh) during the relative displacement between the socket and the residual limb (Simulia, 2012). Moreover, the interaction properties between the socket and residual limb were defined using two different COF. One COF represents the presence of sweat ( μ=0.23 ), whereas the other represents the absence of sweat ( μ=0.37 ) as reported by Ramirez et al. (Ramírez et al., 2015).

### Socket donning

During the donning step, a 0.37 COF was assigned on the interface residual limb–socket. This condition represents the presence of hair and absence of sweat (Lacroix and Ramírez Patiño, 2011; Ramírez et al., 2015; Fuentes et al., 2013). For socket donning, a displacement vector was applied in the most distal zone of the socket (see Figure 2). Each vector was defined uniquely for each subject. This step was carried out in 15 s (Lacroix and Ramírez Patiño, 2011), which represents the duration of the actual donning process. Furthermore, the BC that restricts the displacements and rotations was defined from this step for all models developed (see Figure 2).

### Stabilization

This step was defined to mitigate the dynamic effects and allow soft tissue rearrangement inside the socket. This step was carried out in 5 s while retaining the BC as defined in the previous step. Lacroix and Ramirez (Lacroix and Ramírez Patiño, 2011) previously demonstrated that the model reaches a stable state within this 5 s period, with low residual deformation energy. Therefore, the results corresponding to this stage are not reported, as they do not provide relevant information for the objectives of the present study.

### Gait cycle

After finalizing steps 1 and 2, an external stimulus in these models was defined. These stimuli represent the maximum stress condition for each patient during the gait cycle. A previous study (Ramírez, 2011) was carried out to define the loads for each patient using the inverse dynamic model. These loads were applied in the most distal zone of the socket. The COF was modified for this step, to represent the presence of hair and sweat (COF = 0.23). This step was carried out in 0.1 s, which represents immediate contact during the gait cycle (Lacroix and Ramírez Patiño, 2011).

### Statistical analysis

To determine whether the gluteus zone significantly impacts the stress state in the residual limb–socket interaction, Tukey repeated measures analysis with 95% confidence interval was used. Three treatments and 15 repeats were defined, corresponding to the three models developed and repeats in each patient. This analysis was done for all results obtained (CPRESS, CSHEAR 1, CSHEAR 2, and the total contact area between the socket and the residual limb) in steps 1 and 3.

## Results

Our analysis focused on the residual limb–socket interaction. Given the large deformations of the soft tissue during the simulation process, there may be nodes that deform excessively independently, generating incorrect maximum values for the models. For this reason, a filtering process of the results obtained (CPRESS, CSHEAR1, and CSHEAR2) was included to calculate the real average of the results of interest. In other words, this process allowed us to discriminately obtain nodes whose results do not have outliers values and are different from zero (0). Figure 3 shows a scatter plot for reference patient P6 during donning in model 3; however, this process was performed for all patients. Table 2 shows the results obtained (CPRESS, CSHEAR1, and CSHEAR2) for each model carried out during the donning and gait cycle. Figure 4 shows graphically the variation of the contact stress results obtained for patient P6.

**FIGURE 3:**
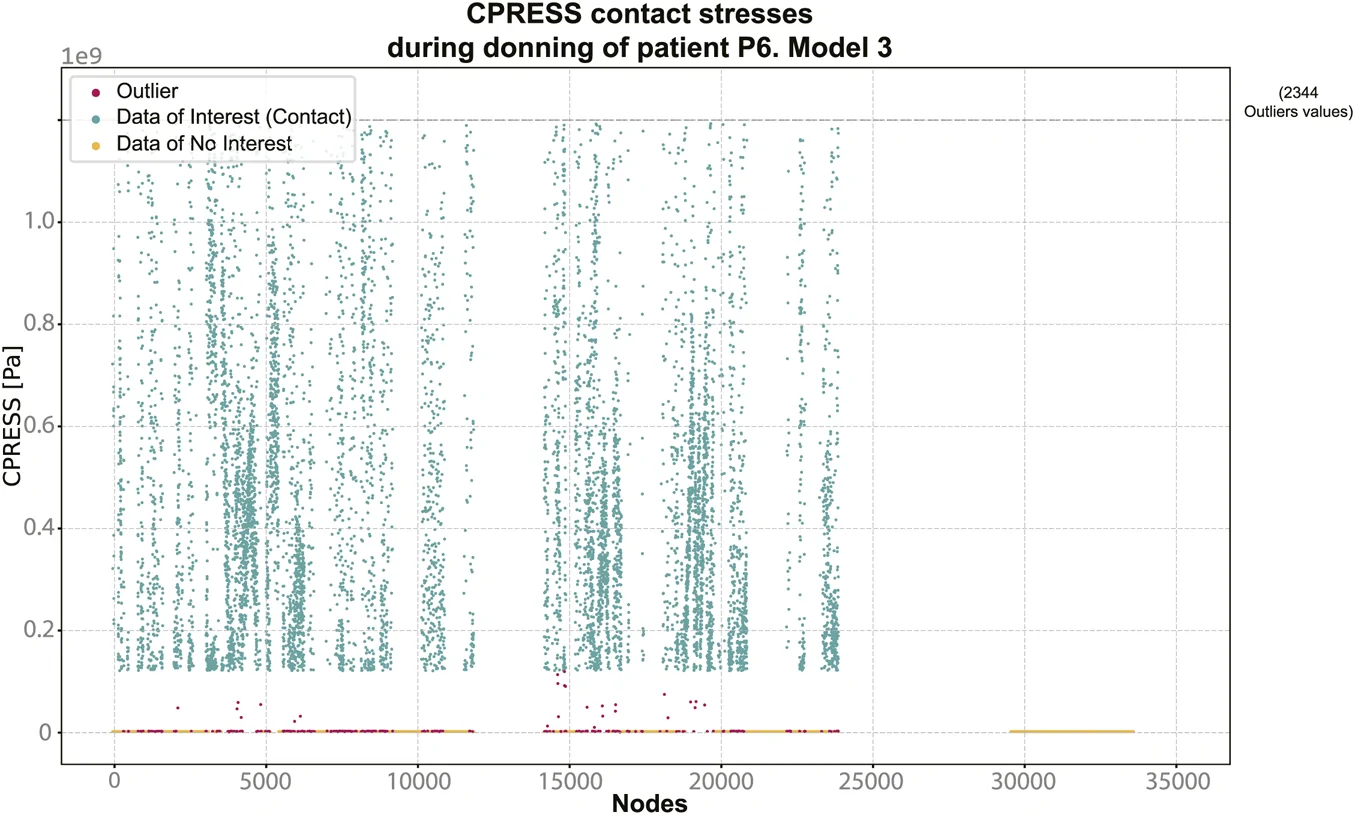
Scatter plot of values for CPRESS stress during the donning process for patient P6 in model type 3. Blue, values of interest (i.e., nodes that come into contact and are not outliers), Yellow, values of no interest (i.e., nodes that do not come into contact), and Magenta, outliers (i.e., nodes that were distorted during the simulation).

**TABLE 2:** CPRESS, CSHEAR1, and CSHEAR2 contact stress values for the 15 patients in each of the simulation conditions analyzed.

Patient	Model	Donning avg (max*)	GC avg (max*)	Donning (max)	GC (max)	Donning (max)	GC (max)
		CPRESS (kPa)	CPRESS (kPa)	CSHEAR1 (kPa)	CSHEAR1 (kPa)	CSHEAR2 (kPa)	CSHEAR2 (kPa)
1	1	12.245 (43.3)	8.779 (43.9)	20.021	11.103	15.297	9.001
	2	16.321 (98.8)	13.368 (98.2)	36.524	14.238	59.962	72.158
	3	17.208 (97.4)	13.002 (99.6)	35.820	19.959	84.532	96.996
2	1	14.790 (54.6)	10.498 (44.0)	55.182	10.309	20.049	8.766
	2	26.566 (199.0)	19.898 (193.8)	91.627	10.789	82.866	10.896
	3	31.098 (246.0)	20.403 (198.4)	104.369	27.745	23.362	63.640
3	1	18.575 (65.1)	14.765 (64.7)	21.673	16.456	21.311	11.668
	2	26.383 (98.2)	25.199 (99.2)	29.824	11.482	37.087	20.786
	3	28.894 (120.0)	30.337 (119.7)	27.807	12.265	39.383	29.066
4	1	13.004 (61.5)	11.986 (57.1)	4.817	7.741	25.733	5.364
	2	25.083 (180.0)	27.084 (219.7)	16.572	38.970	56.559	47.110
	3	28.830 (242.0)	29.816 (249.8)	19.497	52.819	56.719	57.784
5	1	24.900 (598.0)	33.200 (1,000.0)	8.327	26.938	25.682	43.780
	2	35.000 (1,000.0)	35.600 (1,000.0)	18.168	30.004	72.953	32.987
	3	35.500 (1,000.0)	15.200 (200.0)	25.216	26.093	53.101	47.378
6	1	32.500 (998.0)	39.500 (999.0)	13.916	25.780	13.930	8.510
	2	37.700 (998.0)	36.700 (1,000.0)	62.353	36.508	47.113	76.786
	3	39.000 (999.0)	37.000 (1,000.0)	86.920	33.150	51.554	35.068
7	1	34.300 (997.0)	34.100 (998.0)	7.901	5.758	13.980	34.652
	2	31.200 (995.0)	38.000 (1,000.0)	50.856	77.339	20.329	28.413
	3	34.200 (999.0)	38.500 (1,000.0)	54.528	33.264	18.337	36.113
8	1	32.800 (999.0)	34.700 (996.0)	0.241	10.166	14.371	7.156
	2	26.800 (790.0)	25.800 (784.0)	3.595	10.740	12.569	4.090
	3	27.100 (786.0)	26.200 (783.0)	4.019	10.867	13.864	3.740
9	1	35.100 (995.0)	35.900 (999.0)	4.655	5.589	16.571	20.527
	2	38.300 (994.0)	35.600 (999.0)	21.194	16.818	33.719	13.288
	3	41.700 (998.0)	37.700 (998.0)	35.777	18.674	57.919	17.932
10	1	37.600 (992.0)	36.300 (1,000.0)	33.330	58.249	55.286	73.807
	2	42.500 (999.0)	39.600 (1,000.0)	75.517	69.362	19.701	45.490
	3	41.000 (999.0)	42.800 (999.0)	17.000	97.593	15.577	73.621
11	1	34.600 (887.0)	45.500 (918.0)	7.608	4.990	10.402	8.242
	2	35.300 (985.0)	33.800 (979.0)	16.335	10.332	33.531	1.891
	3	38.800 (997.0)	37.500 (999.0)	36.128	25.157	53.548	5.166
12	1	43.400 (988.0)	35.500 (1,000.0)	5.113	13.732	38.551	7.689
	2	43.800 (999.0)	36.900 (999.0)	6.745	5.720	43.987	21.254
	3	43.000 (999.0)	35.500 (1,000.0)	5.776	13.060	40.206	17.376
13	1	33.600 (999.0)	35.600 (997.0)	4.177	13.734	24.378	12.354
	2	36.900 (1,000.0)	37.800 (1,000.0)	33.656	49.338	13.032	47.259
	3	36.500 (1,000.0)	38.400 (999.0)	40.000	60.378	19.960	47.661
14	1	42.600 (997.0)	36.700 (994.0)	10.406	6.022	60.495	4.360
	2	40.500 (998.0)	37.900 (999.0)	23.826	33.961	71.368	45.922
	3	40.700 (999.0)	37.600 (998.0)	69.875	74.753	82.978	31.905
15	1	37.720 (986.0)	33.326 (995.2)	55.425	17.655	27.706	6.977
	2	39.906 (996.0)	39.500 (999.7)	28.932	14.993	19.941	19.621
	3	38.520 (997.0)	37.837 (999.7)	50.000	17.504	31.359	72.804

* These maximum values correspond to the peak CPRESS, after the filtering process.

**FIGURE 4:**
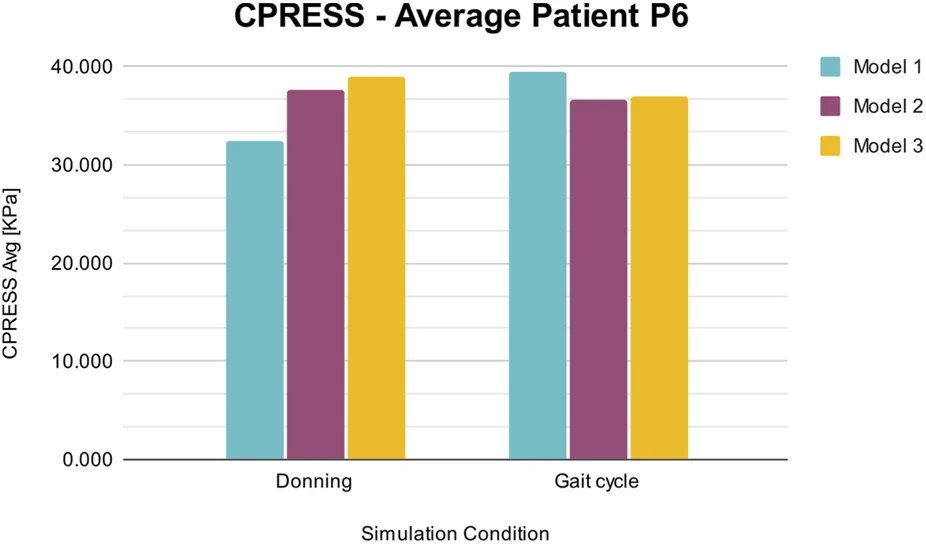
Bar graph for average CPRESS strain during the donning process and gait cycle for patient P6 in model type 3.

As shown in Henao et al. (Henao et al., 2020a), there are seven spatially distributed zones around the residual limb of a PTA, representing pressure tolerant and pressure sensitive zones (see Figure 5) This distribution allows interpretation of whether the stress distribution results of the models are within a suitable zone (pressure tolerant zone) or whether they are in a zone susceptible to skin damage (pressure sensitive zone) and therefore generating soft tissue pain. Table 3 shows in which zone the maximum pressure value (CPRESS) is located during the gait cycle for each patient in each of the developed models, identifying whether the maximum CPRESS is in a pressure tolerant or pressure sensitive zone and how the location of the maximum CPRESS varies depending on the type of model developed.

**FIGURE 5:**
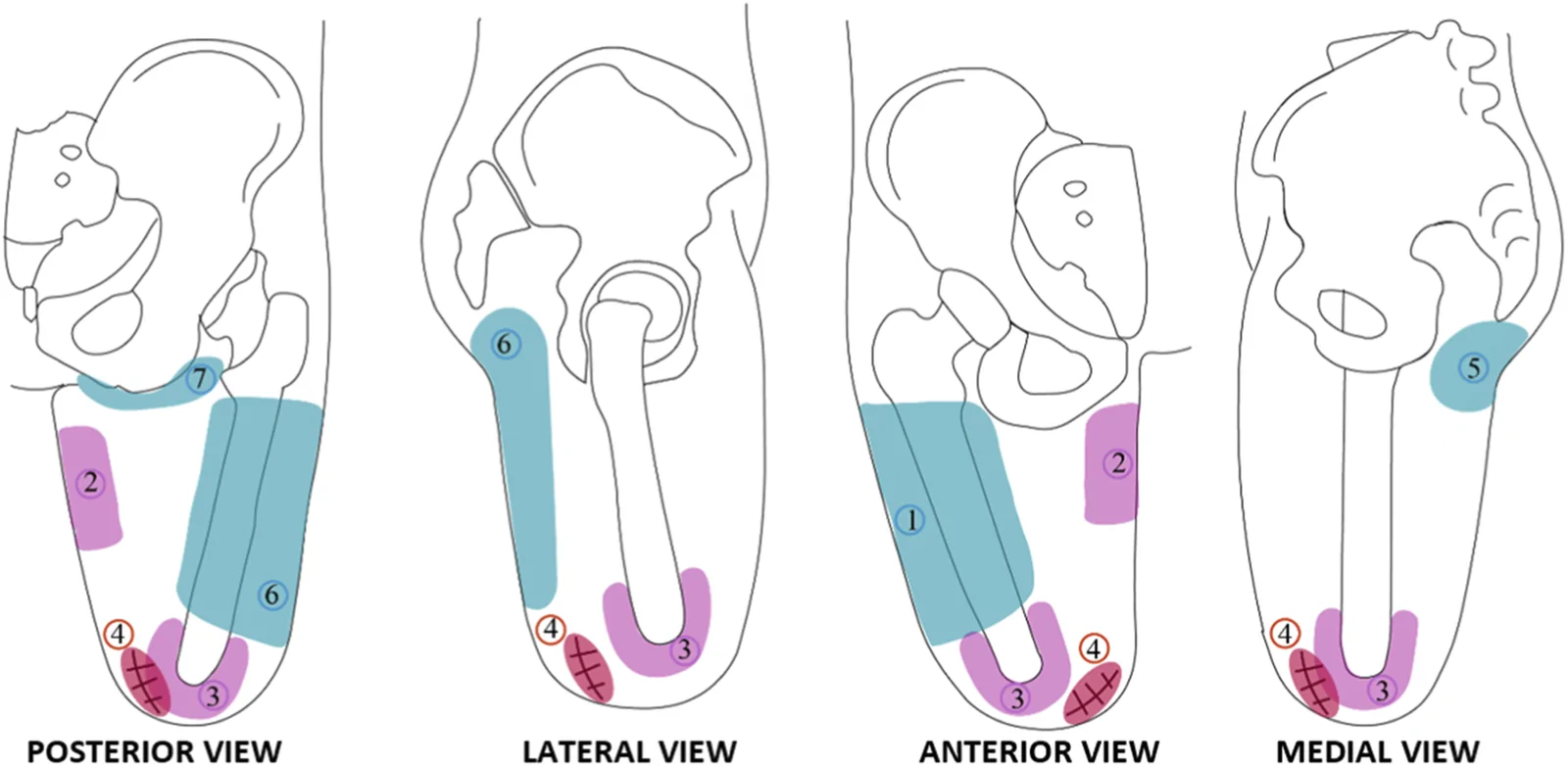
Pressure tolerant zone (blue) and pressure sensitive zone (pink and red) (Henao et al., 2020a).

**TABLE 3:** Maximum contact stress location according to the distribution of pressure zones during the gait cycle of each patient.

Patient	Simulation condition					
	Gait cycle					
	Model 1		Model 2		Model 3	
	Zone	Zone type	Zone	Zone type	Zone	Zone type
P1	Zone 2	Sensible	Zone 1	Tolerant	Zone 1	Tolerant
P2	Zone 1 & zone 2	Tolerant/Sensible	Zone 2	Sensible	Zone 5	Tolerant
P3	Zone 2	Sensible	Zone 1	Tolerant	Zone 1	Tolerant
P4	Zone 4	Sensible	Zone 5	Tolerant	Zone 5	Tolerant
P5	Zone 5	Tolerant	Zone 2	Sensible	Zone 2	Sensible
P6	Zone 1	Tolerant	Zone 2	Sensible	Zone 2	Sensible
P7	Zone 5	Tolerant	Zone 1	Tolerant	Zone 1	Tolerant
P8	Zone 6	Tolerant	Zone 5	Tolerant	Zone 5	Tolerant
P9	Zone 1	Tolerant	Zone 5	Tolerant	Zone 5	Tolerant
P10	Zone 1	Tolerant	Zone 1	Tolerant	Zone 1	Tolerant
P11	Zone 6	Tolerant	Zone 6	Tolerant	Zone 6	Tolerant
P12	Zone 6	Tolerant	Zone 1	Tolerant	Zone 6	Tolerant
P13	Zone 6	Tolerant	Zone 2	Sensible	Zone 2	Sensible
P14	Zone 7	Tolerant	Zone 7	Tolerant	Zone 7	Tolerant
P15	Zone 1	Tolerant	Zone 7	Tolerant	Zone 7	Tolerant

Additionally, Table 4 shows the results for reference patient P3 and how the zones and stress distribution changes between each of the models developed in the donning process and gait cycle.

**TABLE 4:** Variation of CPRESS stress distribution in the posture and gait cycle among the three different models for patient P3.

Model	Gait cycle (CPRESS [Pa])			
	Anterior view	Lateral view	Posterior view	Medial view
M1	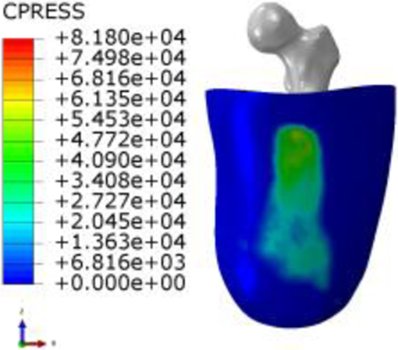	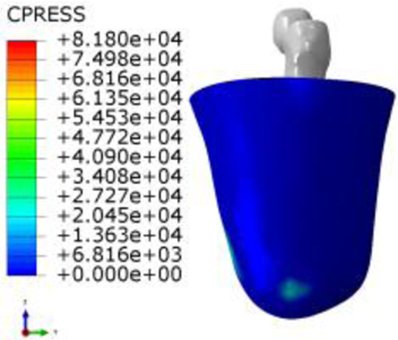	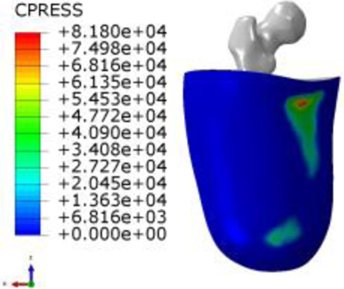	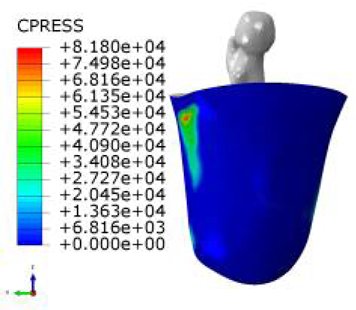
M2	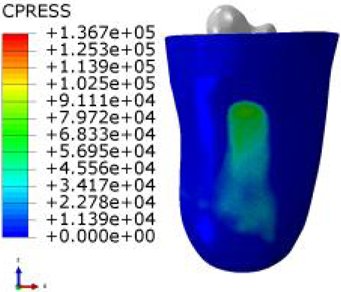	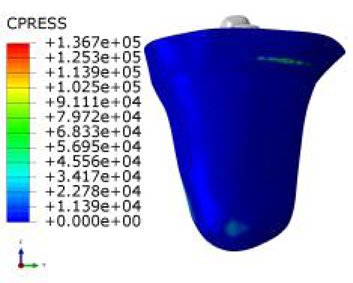	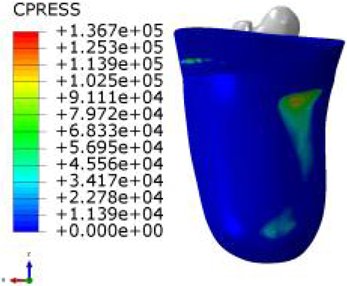	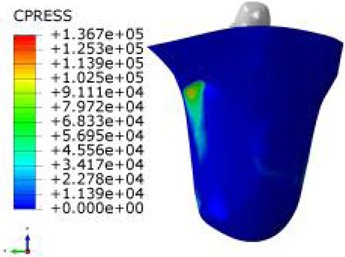
M3	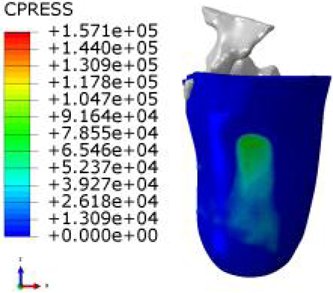	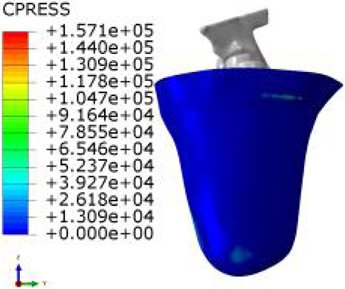	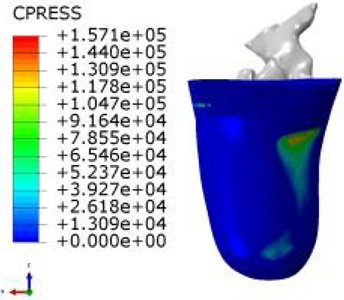	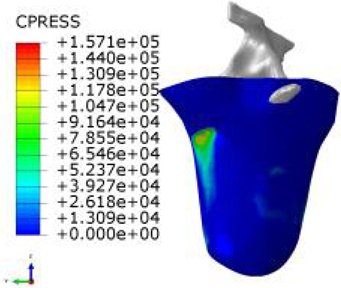
Gait cycle (CPRESS [Pa])				
M1	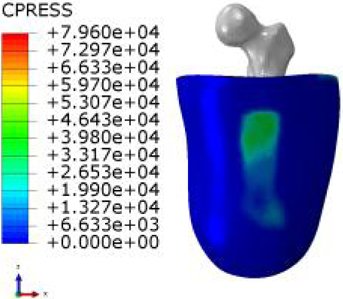	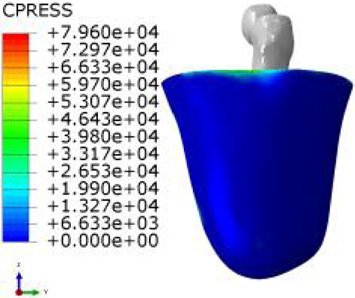	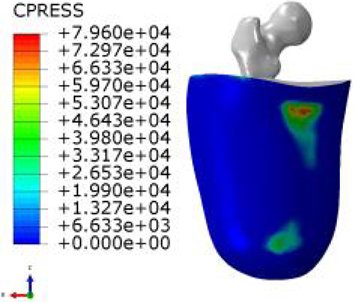	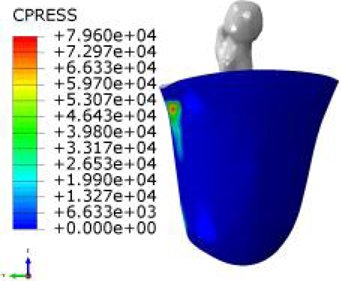
M2	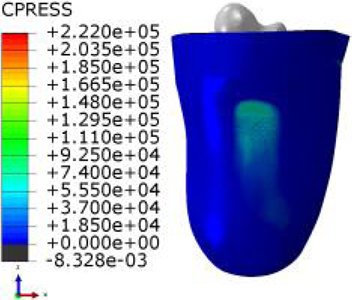	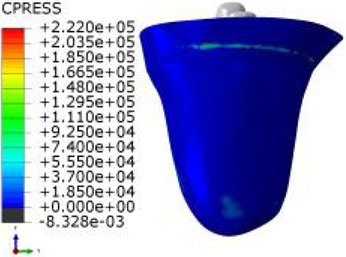	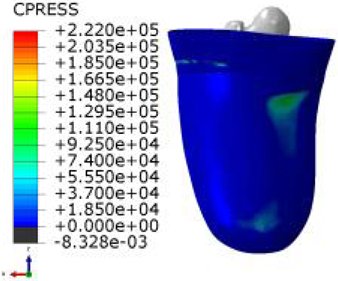	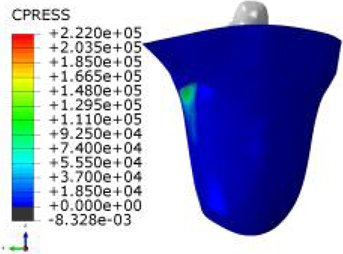
M3	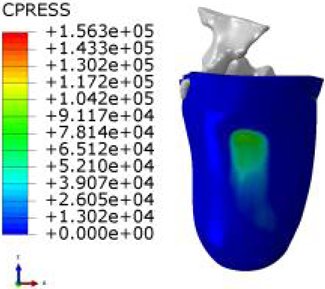	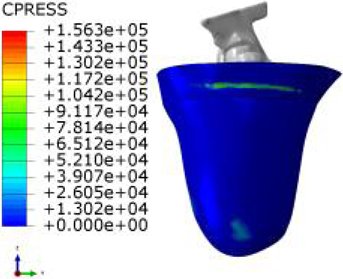	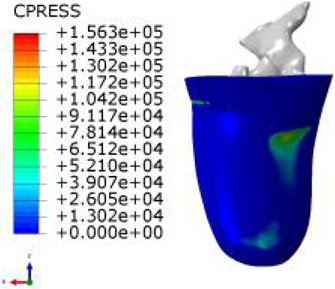	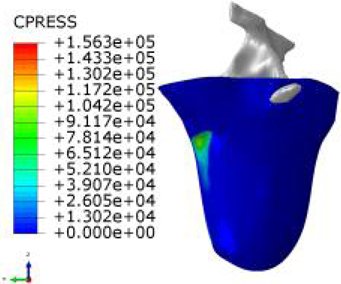

Finally, Table 5 shows the results of the statistical analysis for each of the comparisons evaluated. This technique aimed to determine whether there was a statistically significant difference between the different treatments (type of model). Each of the comparisons should be analyzed independently. Sharing of the same letter between two or more groupings suggests no statistically significant difference between the treatments that share it. For instance, in the comparison made for the CPRESS contact stress during donning, it can be observed in the grouping column that all treatments in this comparison share the letter A, which implies that no statistically significant difference was observed between the three different treatments analyzed.

**TABLE 5:** Results of the different comparisons made with Tukey’s multiple comparison model between the three types of treatments.

Comparisons	Treatments	N	Mean* (kPa)	Grouping
CPRESS-donning	Model 1	15	29.849	A
	Model 2	15	33.803	A
	Model 3	15	32.484	A
CPRESS-gait cycle	Model 1	15	29.757	B
	Model 2	15	32.183	B
	Model 3	15	31.853	B
CSHEAR1-donning	Model 1	15	13.519	C
	Model 2	15	83.351	C
	Model 3	15	99.048	C
CSHEAR1-gait cycle	Model 1	15	45.615	D
	Model 2	15	65.373	D
	Model 3	15	53.552	D
CSHEAR2-donning	Model 1	15	44.916	E
	Model 2	15	65.493	E
	Model 3	15	64.981	E
CSHEAR2-gait cycle	Model 1	15	67.524	F
	Model 2	15	83.197	F
	Model 3	15	66.416	F

* This statistical mean has been calculated using the maximum (CSHEAR1 and CSHEAR2) and the average contact stress (CPRESS) values presented at Table 2 based on Tukey’s model.

## Discussion

The current study developed 45 numerical models of PTA corresponding to the 15 patients included herein. This study focused on the analysis of the interaction between the residual limb and the socket.

Table 2 shows the results of the mean contact values in the simulation conditions of interest. Accordingly, our findings showed similar behavior in terms of mean CPRESS values for each of the types of models developed. Moreover, it is evident that models 2 and 3 tended to have higher mean contact stress values relative to model 1. After a detailed observation, model 3 showed slightly higher mean contact stresses than model 2, although in some cases this variation did not exist and the mean contact stresses remained constant. The behavior of the described results can be attributed to two reasons: first, including the gluteal zone in the models allows for the identification of results that could not be observed previously in the type 1 models. For instance, in the lateral zone of patient P3 in Table 4, we can observed that the two simulation conditions in models 2 and 3 had a stress distribution in the gluteal zone that could not be observed in model 1. Moreover, models 2 and 3 reflect the behavior in terms of stress distribution expected during the design process of prosthetic systems for PTA. Accordingly, we observed that the internal geometries that force the socket to generate pressures in areas identified as tolerant simultaneously release pressures in sensitive areas of the residual limb. As can be seen in Table 3, the location of the maximum stress zone changes depending on the type of model analyzed in 80% of the patients. For example, for patient P3, the maximum stress in model 1 is located in zone 2, whereas that in models 2 and 3 is located in zone 1. While for patient 14 maximum stress remains at zone 7.

Table 3 shows a variation of the location of the maximum contact stress between the three models for all patients. However, this does not necessarily imply a change in the type of zone (i.e., a change from one zone that is tolerant to a different zone that is also tolerant). There are models in which both the location and zone type change. We observed that the maximum contact stress in model 1 was located in a tolerant zone, whereas that in models 2 or 3 was located in a sensitive zone for the same patient. This suggests that including the gluteal zone in this type of model can influence the location of the maximum stress. Apart from showing the impact of including the gluteal area in this type of model, this result also indicated poor socket design for patients in whom this behavior occurs given that the soft tissue areas where the maximum contact stresses are located are sensitive areas, which is a completely different response to what is required with a properly designed socket.

To further characterize the effect of incorporating the additional proximal anatomy, Table 6 summarizes the changes observed between M1 and M3 in the location and classification of the maximum contact stress zone across the 15 patients. Although the inclusion of the gluteal region and pelvic bone did not significantly change the mean contact stress magnitude, the maximum-stress zone changed its anatomical location in 12 of the 15 patients (80%). However, the type of zone associated with the maximum stress changed in only 6 patients (40.0%), with an equal number of patients showing a shift toward a more tolerant or more sensitive zone. Importantly, the proportion of patients for whom the maximum stress was located in a tolerant zone remained unchanged (80%) across M1and M3. These results indicate that the incorporation of additional proximal anatomy primarily affects the spatial distribution of contact stresses rather than the overall magnitude or the general classification of the zone supporting the maximum stress.

**TABLE 6:** Changes in the anatomical location and tolerance classification of maximum-stress zones across gait-cycle simulations.

Metric (M1 vs. M3, gait cycle, n = 15)	Result
Patients whose maximum-stress zone changed anatomical location (of 7 zones)	12/15 (80%)
Patients whose zone type changed (tolerant ↔ sensitive)	6/15 (40.0%), symmetric: 3 became more tolerant, 3 became more sensitive
Proportion of patients with maximum stress in a tolerant zone, per model	80% in M1and M3 (unchanged)

Table 4 shows that there was variation in the distribution of the state of contact stresses in the residual limb among the three different models for patient P3. A similar behavior was noted in the 80% of the patients analyzed. In the lateral view during the two simulation conditions of interest (donning and gait cycle), we observed that models 2 and 3 had contact stress distribution zones that were not identified in model 1. Furthermore, in the posterior view during the two simulation conditions, with model 1 as a reference, we identified a zone where contact stresses concentrate. This zone decreased in intensity in models 2 and 3 with the appearance of a new stress zone, which indicates that including the gluteal zone in this type of models provides additional information. This behavior suggests that including the gluteal zone in this type of models is consistent with the purpose of designing sockets for PTA due to the design process of the sockets, which aims to concentrate stresses in areas classified as tolerant in the residual limb, consequently releasing pressures in areas classified as sensitive.

Table 5 shows no significant statistical difference in the mean CPRESS contact stresses found between the three types of models. This implies that developing models of PTA that do not include the gluteal zone and pelvic bone may provide mean contact stresses representative of the residual limb and the socket. However, as observed in the current study, not including the gluteal zone and pelvic bone in this type of model prevents us from observing the actual contact stress distribution zones on the residual limb surface. This suggests that including the gluteal zone in this type of model, although it does not provide different mean results in terms of magnitude compared to models that do not include it, allows for the more accurate identification of where the highest contact stresses are concentrated in the residual limb and what type of zone (tolerant or sensitive) is supporting the greatest load, thereby allowing for more accurate decisions during the design process of sockets for PTA.

As reported in Dickinson et al. (Dickinson et al., 2017), only a handful of studies have developed dynamic numerical models for PTA, which implies that a comparison of results with other authors would not be comprehensive. However, Table 2 shows the maximum values obtained for the CPRESS contact stresses in each model performed for the 15 patients, with values ranging from 43 to 1,000 kPa. Comparing the values of the maximum stresses obtained in this study with those reported by Zhang et al. (Zhang et al., 2013) (50 and 105 kPa), Ramírez et al. (Lacroix and Ramírez Patiño, 2011) (20 and 800 kPa), and Henao et al. (Henao et al., 2020a) (80 and 710 kPa), it can be observed that the maximum results obtained in this study are of equal magnitude to those previously reported by these authors.

The range of maximum values obtained is very extensive, which implies considerably variability in the data. This can be explained by Ramírez et al. (Ramírez, 2011) who showed that the model developed for each patient is unique due to the characteristics that cannot be systematically replicated, such as the geometries of the residual limb, the orientation of the residual bone with respect to the residual soft tissue, the relation of the residual limb volume with the socket, and the loads due to the gait cycle of each patient.

Furthermore, the study by Dickinson et al. (Dickinson et al., 2017) showed that throughout history, no numerical model studies for PTA that include the pelvic bone have been reported. However, Heesewijk et al. (van Heesewijk et al., 2018) reported a first approximation of the implications of including the pelvic bone in numerical models of PTA, where a peak in normal contact stresses of 364 KPa is reported. Nevertheless, this first approximation has a simulation process that is far from reality and differs from the simulation processes reported in previous studies (i.e., the simulation process considers applying a load in the distal area of the socket and not a displacement vector to represent the donning process of the socket; obtaining the geometry of the residual limb from medical images obtained in supine position, which implies that the soft tissue is deformed at the time of reconstruction; and ensuring that the socket has an internal geometry exactly the same as the external geometry of the residual limb, which implies that it does not consider the pressure zones purposely made by the prosthetists to release pressures in defined areas of the residual limb), which may generate inaccurate results.

## Conclusion

Including the pelvic bone and the gluteal zone in models (M1, M2, M3) of people with transfemoral amputation had not statistically significant difference on the magnitude of the mean contact stress (CPRESS, CSHEAR1, and CSHEAR2) in the interaction between the residual limb and the socket during the gait cycle. However, it did influence the distribution of the stress state. After comparing the M1 vs. M2 and M2 vs. M3 models, a significant difference in stress distribution was observed between M1 and M2, with differences identified in 80% of the models. In contrast, no significant difference in stress distribution was observed between M2 and M3, with differences identified in only 13.3% of the models. These results indicate that the inclusion of the pelvic bone had no significant effect on the distribution of the contact stress state and the addition of the gluteal zone only, resulted in a significant difference in the stress state distribution.

The incorporation of additional proximal geometries, such as the gluteal region and pelvic bone, may not be necessary in simplified FEA models when the objective is to evaluate the mechanical response of a given patient while maintaining the same socket configuration, particularly when their inclusion does not produce significant changes in the quantities of interest and entails an increase in model complexity and computational cost. These findings are intended to assess the relevance of increasing anatomical model fidelity and should not be interpreted as an evaluation of different socket designs or the biomechanical effect of ischial support.

These findings should not be used to support direct clinical decision-making or to replace patient-specific clinical assessment. Rather, the results provide engineering-level information that may support the design and development of prosthetic sockets by helping determine the level of anatomical detail required in numerical models while balancing mechanical fidelity and computational cost.

For the conditions investigated in this study, the pelvic bone does not need to be explicitly modeled when the primary outcomes of interest are the mean contact stresses, as its inclusion increases model complexity and computational cost without providing a significant benefit in these quantities. In contrast, the gluteal region may be relevant when the location of the maximum contact stress is of interest. These conclusions apply to the specific conditions investigated here: a single loading instant, single-bulk tissue representation, distal loading, and no imposed ischial contact.

### Limitations and future work

Several limitations should be considered when interpreting the results of the present study. First, the geometry of the gluteal region represents an approximation based on anatomical reconstruction. This limitation arises from the inherent constraints of the medical imaging data used to reconstruct the residual limb, as the gluteal region may be deformed during image acquisition and therefore may not represent its unloaded anatomical configuration. Second, the boundary conditions and applied loads were derived from a generic inverse kinematics model adapted to each individual at the stance phase in which the ground reaction force reaches its maximum value rather than directly from experimental measurements obtained through a gait analysis laboratory. Consequently, patient-specific variations in loading and movement conditions may not be fully represented. Third, the present study does not explicitly evaluate the biomechanical effect of ischial support, as different socket configurations were not compared for the same patient. These limitations should be considered when interpreting the mechanical response predicted by the models.

Based on the objective of the present study, the numerical model included several simplifications. The bone was modeled as a homogeneous material without differentiating between cortical and trabecular bone. Similarly, the soft tissues were not discriminated into individual anatomical layers. The models do not incorporate additional anatomical structures such as articular cartilage. These assumptions were made to maintain an appropriate balance between the complexity of the model and the objective of the study. Although the numerical models included a stabilization step, the strain energy values were monitored and verified to ensure that the model reached a stable condition; however, these values are not reported in the manuscript because they were not considered relevant to the main objective of the study. The numerical results were not experimentally validated. Instead, the resulting stress states were compared with values reported in the literature for similar numerical models. This comparison was used to assess whether the magnitude and distribution of the predicted stresses were consistent with previously reported results.

Finally, an additional limitation is that the assessment of displacement between the defined zones was based on a visual classification of the displacement pattern rather than on a normalized quantitative metric. Although this approach allowed the displacement behavior to be characterized according to the objective of the present study, it may introduce a degree of subjectivity in the classification. Future work should therefore investigate quantitative and normalized strategies to objectively characterize displacement between zones and facilitate comparisons across patients and models.

Future work should investigate the influence of increasing the anatomical and biomechanical fidelity of the models. In particular, more detailed representations of the soft tissues could be developed by incorporating distinct layers of skin, adipose tissue, and muscle. Additionally, improved discretization strategies should be evaluated to obtain more stable meshes, including the use of hexahedral elements where the geometry permits. The incorporation of additional anatomical structures, such as the articular cartilage of the acetabulum, could also provide a more comprehensive representation of the proximal anatomy. Furthermore, future studies should consider loading and motion conditions derived directly from experimental gait analysis measurements. Finally, dedicated studies comparing different socket configurations for the same patients would be required to specifically evaluate the biomechanical role of ischial support and its influence on residual limb–socket load transfer. These extensions would allow assessment of whether the conclusions of the present study remain valid as the anatomical and biomechanical fidelity of the models is progressively increased.

## Data Availability

The raw data supporting the conclusions of this article will be made available by the authors, without undue reservation.

## Footnotes

1 LLA Lower limb amputation.

2 QOL Quality of life.

3 FEM Finite element method.

4 PTA People with transfemoral amputation.

5 COF Coefficients of friction.

6 BC Boundary conditions.
